# Laccase-Mediator System Using a Natural Mediator as a Whitening Agent for the Decolorization of Melanin

**DOI:** 10.3390/polym13213671

**Published:** 2021-10-25

**Authors:** Saerom Park, Dahun Jung, Hyejin Do, Jonghyeon Yun, Dongjun Lee, Soeun Hwang, Sang Hyun Lee

**Affiliations:** Department of Biological Engineering, Konkuk University, Seoul 05029, Korea; angel4y@naver.com (S.P.); 1wjdekgns@naver.com (D.J.); dkssud12370@naver.com (H.D.); dbsjh6158@gmail.com (J.Y.); dlehdwns1213@gmail.com (D.L.); thdmsdlrh@naver.com (S.H.)

**Keywords:** laccase, melanin, decolorization, natural mediators

## Abstract

In this study, a laccase-mediator system (LMS) using a natural mediator was developed as a whitening agent for melanin decolorization. Seven natural mediators were used to replace synthetic mediators and successfully overcome the low redox potential of laccase and limited access of melanin to the active site of laccase. The melanin decolorization activity of laccases from *Trametes versicolor* (lacT) and *Myceliophthora thermophila* (lacM) was significantly enhanced using natural mediators including acetosyringone, syringaldehyde, and acetovanillone, which showed low cytotoxicity. The methoxy and ketone groups of natural mediators play an important role in melanin decolorization. The specificity constants of lacT and lacM for melanin decolorization were enhanced by 247 and 334, respectively, when acetosyringone was used as a mediator. LMS using lacM and acetosyringone could also decolorize the melanin present in the cellulose hydrogel film, which mimics the skin condition. Furthermore, LMS could decolorize not only synthetic eumelanin analogs prepared by the oxidation of tyrosine but also natural melanin produced by melanoma cells.

## 1. Introduction

Laccases (EC 1.10.3.2, benzenediol: dioxygen oxidoreductases) are multicopper proteins that catalyze the oxidation of various phenolic and non-phenolic compounds via a radical-catalyzed reaction mechanism by the reduction of molecular oxygen [1,2]. Laccases have been used as biocatalysts for biodegradation processes, such as the bioremediation of dyes [3,4], pharmaceuticals [5,6], and herbicides [7], and delignification [8,9,10]. Laccases have also been used to catalyze the polymerization of dye precursors and organic compounds [11]. In particular, their attractive properties, such as low substrate specificity, the use of oxygen as the final electron acceptor, generation of water as a by-product, and no demand (or no production) of peroxides, make them interesting in biotechnological and environmental fields [1,11,12].

Four copper ions at the active site are involved in the catalytic activity of laccase. “Blue” copper (T1 site) oxidizes the substrate, and the trinuclear copper cluster (T2/T3) receives the electrons from the T1 site to reduce the molecular oxygen [1,12,13]. In particular, the redox potential of the T1 site Cu is considered as a major factor in determining the catalytic ability of laccases [14]. Laccases possess a relatively low redox potential (0.4–0.8 V) compared to ligninolytic peroxidases (over 1 V) such as manganese peroxidase and lignin peroxidase. Laccases cannot directly oxidize non-phenolic substrates with redox potential above 1.3 V [13,14]. Therefore, to overcome the limitations of laccase, laccase-mediator systems (LMS) using small molecular compounds, such as 2,2′-azinobis(3-ethylbenzthiazoline-6-sulphonate) (ABTS), 1-hydroxybenzotriazole (HOBt), violuric acid (VLA), *N*-hydroxyphthalimide (HPI), *N*-hydroxyacetanilide (NHA), and TEMPO, which act as redox mediators, have been suggested [15,16,17].

These mediators permit the oxidation of bulky compounds via different oxidation routes. The laccase-ABTS system oxidizes substrates by generating a cationic ABTS radical via an electron transfer (ET) mechanism. LMSs with HOBt, VLA, HPI, or NHA produce nitroxyl radicals via the hydrogen atom transfer (HAT) mechanism [1,12,17]. Furthermore, mediators such as TEMPO and its analogs react via ionic pathways to generate oxoammonium ions [1,12,18]. The use of these mediators can oxidize a wide range of compounds in various applications, such as dye degradation [3,4], drug degradation [5,6], and lignin degradation [8,9,10]. Nevertheless, the applications of synthetic mediators in industrial fields have been limited due to their potential toxicity, high cost, and enzyme inactivation effect. Recently, lignin-derived phenolic molecules as natural mediators (e.g., syringaldehyde, acetosyringone, vanillin, acetovanillone, methylvanillate, ferulic acid, sinapic acid, *p*-coumaric acid, etc.) have been studied to replace synthetic mediators [1,12]. The advantages of natural mediators are low cost and low toxicity because they are obtained from natural and renewable sources [19].

Melanin is a group of natural pigments produced by melanogenesis through the oxidative polymerization of tyrosine by melanocytes. Natural melanin can be classified into five categories of eumelanin, pheomelanin, allomelanin, pyomelanin, and neuromelanin [20]. Recently, various medical and electrochemical applications using melanin or melanin precursors have been studied [20,21]. The human skin color is mostly determined by the presence of melanin. In the cosmetic industry, the direct depigmentation of melanin using enzymes has been proposed for the development of skin-whitening agents. Several peroxidases have been studied to decolorize melanin. Woo et al. showed that synthetic melanin can be directly decolorized by lignin peroxidase from *P. chrysosporium* [22]. The Keneko and Mohorčič groups also reported the enzymatic decolorization of melanin by manganese peroxidase isolated from fungi (*Sporotrichum pruinosum* and *Phlebia radiata*) [23,24]. Kim et al. reported that crude enzyme mixtures containing manganese peroxidase, lignin peroxidase, and laccase showed melanin depigmentation activity [25]. When peroxidases decolorize melanin, they require hydrogen peroxide (H_2_O_2_) as a cofactor that can irritate the skin. Thus, to reduce the usage of H_2_O_2_, glucose oxidase or laccase was introduced into the enzyme combination system [26,27]. Laccases can decolorize melanin without the use of hydrogen peroxide. Khammuang and Sarnthima reported that laccase from *Lentinus polychrous* Lév showed melanin decolorization activity using ABTS, vanillin, and vanillic acid as mediators [28].

In this study, LMS using a natural mediator was developed as a whitening agent for melanin decolorization. Various lignin-derived phenolic molecules have been tested as mediators of melanin decolorization to replace synthetic mediators. The effect of mediator concentration and pH on melanin decolorization in LMS using commercially available laccases from *Trametes versicolor* and *Myceliophthora thermophila* was investigated, and the cytotoxicity of natural mediators was also investigated. The enhancing effect of natural mediators was quantitatively analyzed by a kinetic study of the melanin decolorization reaction using LMS. Furthermore, the decolorization of melanin in the cellulose hydrogel film, which mimics the skin condition, and the decolorization of natural melanin produced by melanoma cells was also studied.

## 2. Materials and Methods

### 2.1. Materials

Laccases from *Trametes versicolor* (lacT), laccase from *Myceliophthora thermophila* (lacM), synthetic melanin, acetosyringone, syringaldehyde, vanillin, *p*-coumaric acid, acetovanillone, vanillic acid, vanillyl alcohol, 1-hydroxybenzotriazole hydrate (HOBt), sodium phosphate dibasic, penicillin, streptomycin, phosphate-buffered saline, neutral red (NR), microcrystalline cellulose (MCC), and 1-ethyl-3-methylimidazolium acetate ([Emim][Ac]) were purchased from Sigma-Aldrich (St Louis, MO, USA). Citric acid was obtained from Junsei (Tokyo, Japan). Trypsin-EDTA, fetal bovine serum, and DMEM were obtained from Thermo Fisher Scientific (Waltham, MA, USA). All chemicals used in this study were of analytical grade and were used without further purification.

### 2.2. Melanin Decolorization by LMS

The saturated melanin solution (1.4 mg/mL) was prepared by the dissolution of 3 mg synthetic melanin in 1.3 mL of 10 mM NaOH. The solution was centrifuged at 8500 rpm for 5 min to remove the undissolved melanin, and the supernatant was diluted with 0.1 M citric acid phosphate buffer (pH 3, 4, 5, 5.5, 6, or 7) and used as a substrate solution for LMS. The concentration of melanin in the substrate solution was 63 μg/mL and spectrophotometrically confirmed at 475 nm. The 0.8 mL of melanin substrate solution was mixed with 0.1 mL mediator solution (0–1 mM) in a 1.5 mL Eppendorf tube. The melanin decolorization reaction was initiated by adding 0.1 mL of laccase solution (15.8 μg (0.6 U) lacT or 19.2 μg (1.8 U) lacM) to a mixture of melanin and the mediator at 25 °C in a shaking water bath at 120 rpm. After the reaction, the reaction mixture was centrifuged, and the absorbance of the supernatant was measured at 475 nm. The decolorization yield (%) was calculated using the following equation:

Decolorization (%) = (A_0_ − A_t_)/A_0_ × 100, (1)

where A_0_ is the absorbance of the melanin solution before the decolorization reaction and A_t_ is the absorbance of the melanin solution after the decolorization reaction.

The protein content of the laccase solution was determined by the BCA method. One unit (U) corresponds to the amount of laccase that converts 1 μmol of ABTS per minute at pH 5.5 and 25 °C.

### 2.3. Kinetic Study of Melanin Decolorization by LMS

To determine the kinetic constants for the melanin decolorization reaction by LMS, the initial rates of laccase (0.6 U lacT or 1.8 U lacM) with or without 0.1 mM acetosyringone were measured using various melanin concentrations (0–420 μg/mL). The melanin solution was prepared by diluting completely dissolved melanin (1 mg/mL) in 10 mM NaOH. The kinetic constants were obtained from the Michaelis–Menten equation using the non-linear regression function of SigmaPlot 12 (Systat Software, San Jose, CA, USA).

### 2.4. Cytotoxicity of Natural Mediators

The B16F10 melanoma cell line (Korea Cell Line Bank, Seoul, Korea) was used to determine the cytotoxicity of natural mediators for LMS. A neutral red (NR) assay was performed to measure the cytotoxicity of the mediators [29]. NR measures the viability of live cell lysosomes. Melanoma cells with a concentration of 3 × 10^4^ cells were dispensed into each well of a 96-well plate. After 24 h of cultivation, the cells were treated with natural mediators (1, 2, 5, 10, 22, and 46 mM). After additional cultivation for 2 days, the cells were treated with 50 μg/mL NR solution dissolved in DMEM and incubated for 3 h. After removing the supernatant through suction, an NR desorb solution (1% glacial acetic acid, 49% ethanol, and 50% distilled water) was used for color extraction. After the extraction process, the change in absorbance was measured at 540 nm.

### 2.5. Preparation and Decolorization of the Melanin/Cellulose Hydrogel Film

To prepare the melanin/cellulose film, 0.5% (*w*/*v*) synthetic melanin was dissolved in [Emim][Ac] under ultrasound irradiation for 10 min. The melanin solution was centrifuged at 8500 rpm for 20 min to remove insoluble melanin, and then 7 wt % of cellulose was dissolved in the supernatant at 100 °C for 2 h with stirring. The mixture solution was cast on a glass slide to a thickness of 0.3 mm using an applicator/1117 (Mitutoyo Corp., Kawasaki, Japan), and dissolved melanin and cellulose were regenerated with distilled water. The prepared film was washed with 0.1 M citric acid phosphate buffer (pH 5.5) until no absorbance of [Emim][Ac] was measured at 211 nm. The melanin/cellulose hydrogel film was stored in 0.1 M citric acid phosphate buffer (pH 5.5) until further use.

To measure the decolorization activity of LMS for the melanin/cellulose film, the prepared hydrogel film was cut into a 1 × 2 cm sheet. The hydrogel film was immersed in 4 mL of 0.1 M citric acid phosphate buffer (pH 5.5); subsequently, 0.5 mL of 1 mM acetosyringone and 0.5 mL of lacM solution (2.5 U) were added to the buffer. The decolorization reaction was carried out in a water bath with shaking at 120 rpm and 25 °C for 3 h. After the reaction, the film was washed with distilled water and attached to the inner side of the cuvette to measure the change in the spectra in the range of 400–800 nm using a UV/Vis spectrophotometer. Control reactions without lacM or mediators were also conducted under the same conditions. The release of melanin from the film or color change of the melanin/cellulose film was not detected under the reaction conditions. Furthermore, the change in color parameters (L*, a*, and b* values) of the melanin/cellulose film after the decolorization reaction by LMS was also recorded using a colorimeter (KONICA MINOLTA, Tokyo, Japan). The ΔL (metric lightness difference), ∆E (total color difference), YI (yellowness index), and WI (whiteness index) values were obtained using the following equations [30,31,32]:

ΔL = L_after_ − L_before_, (2)

ΔE = [(ΔL)^2^ + (a_after_ − a_before_)^2^ + (b_after_ − b_before_)^2^]^0.5^, (3)

YI = (142.86 × b*)/L*,(4)

WI = 100 − [(100 − L*)^2^ + a*^2^ + b*^2^]^0.5^, (5)

where L_after_, a_after_, b_after_, L_before_, a_before_, and b_before_ are the mean color values after and before the decolorization reaction, respectively.

### 2.6. Preparation of Natural Melanin

Natural melanin was obtained from B16F10 melanoma cells. The cells were treated with alpha-melanocyte-stimulating hormone to produce melanin. After 4 days of incubation, the cells were captured using trypsin-EDTA and sonicated for 10 min. The supernatant was obtained by centrifugation at 8000 rpm for 10 min and then adjusted to pH 1.5 using 6 M HCl. The solution was boiled at 100 °C for 4 h to hydrolyze the residual protein fractions. The solution containing natural melanin was washed with acetone, followed by chloroform and ethanol, and then washed with deionized water to eliminate residues, such as cells, media components, and protein fractions [33,34]. All washing processes were performed more than twice. Finally, natural melanin was obtained by freeze-drying and used as a substrate for LMS.

## 3. Results and Discussion

### 3.1. The Effect of Mediators on the Melanin Decolorization by LMS

**Figure 1:**
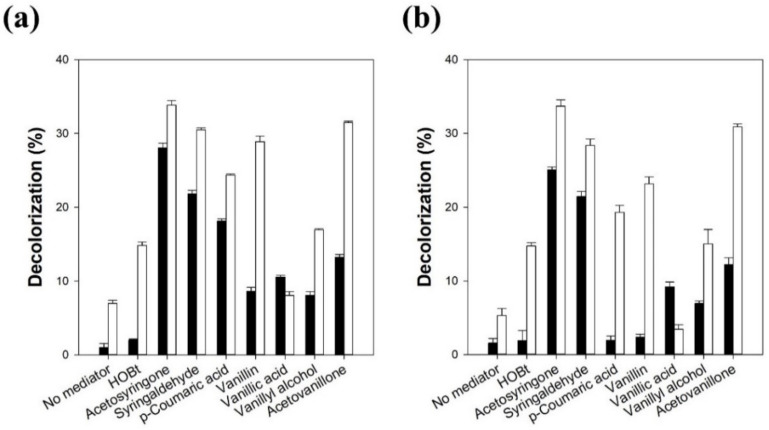
The effect of mediators on the melanin decolorization by LMS using laccase from *T. versicolor* (**a**) and *M. thermophila* (**b**). Black and white bars represent 5 h and 24 h reactions, respectively. Reaction conditions: 50 μg/mL melanin, 0.1 mM mediator, and 0.1 M citric acid phosphate buffer (pH 5.5) at 25 °C.

The effect of various mediators on the melanin decolorization reaction by LMS was investigated using two laccases from *T. versicolor* (lacT) and *M. thermophila* (lacM) (Figure 1).

When lacT was used without a mediator for melanin decolorization, the decolorization yield was only 1% after 5 h of reaction. When HOBt was used as a mediator for lacT, the decolorization yield was slightly enhanced to 2% after 5 h of reaction. The use of various synthetic mediators, such as HOBt, ABTS, VLA, and TEMPO, in the laccase-catalyzed oxidation of phenolic or non-phenolic compounds significantly enhanced the reaction rates [10,15]. When the access of target compounds into the active site of laccase is limited by their steric hindrance, mediator radicals formed by laccase can efficiently oxidize the target compounds by the electron transfer or hydrogen atom transfer mechanism [12]. HOBt is one of the most commonly used synthetic mediators in LMS due to its high redox potential (1.1 V) [6]. However, HOBt is not a good cosmetic ingredient because of its potential cell toxicity and ability to inactivate laccase. Thus, we selected seven natural mediators, acetosyringone, syringaldehyde, *p*-coumaric acid, vanillin, vanillic acid, vanillyl alcohol, and acetovanillone, for the melanin decolorization reaction by LMS. Interestingly, all of the natural mediators act as more efficient mediators than HOBt for melanin decolorization by lacT. When acetosyringone, syringaldehyde, and *p*-coumaric acid were used, the decolorization yields were 28%, 22%, and 18%, respectively, after 5 h of reaction. These results clearly demonstrate the usefulness of natural mediators for the melanin decolorization reaction by LMS. The mediators in the LMS are oxidized to mediator radicals by laccase, and the mediator radicals induce the oxidation and decolorization of melanin. When lacT was used without a mediator for melanin decolorization during a sufficient reaction time, which could reach the equilibrium state, the decolorization yield was 7% after 24 h of reaction. The natural mediators, except vanillic acid, act as more efficient mediators than HOBt for melanin decolorization by lacT after 24 h of reaction. The decolorization yield after 24 h reaction using vanillic acid as a mediator was lower than that after 5 h of reaction. This may be caused by the low stability of the oxidized radical form of vanillic acid. When acetosyringone, syringaldehyde, and acetovanillone were used, the decolorization yields were 34%, 30%, and 31%, respectively, after a 24 h reaction. *p*-Coumaric acid was more efficient in enhancing the initial reaction rate than acetovanillone, while acetovanillone induced a higher decolorization yield at the equilibrium state than *p*-coumaric acid.

The effect of the mediator on the decolorization reaction by LMS using lacM was also very similar to that obtained by LMS using lacT. When lacM was used without a mediator for melanin decolorization, the decolorization yield was only 2% after 5 h of reaction. HOBt as a mediator for lacM did not enhance the decolorization yield during the 5 h reaction. All of the natural mediators, except *p*-coumaric acid and vanillin, acted as efficient mediators of melanin decolorization by lacM. When acetosyringone and syringaldehyde were used, the decolorization yields were 25% and 22%, respectively, after 5 h of reaction. *p*-Coumaric acid and vanillin were used as efficient mediators for lacT, but they could not efficiently enhance the decolorization rate in LMS using lacM. This may be caused by the lower substrate specificity of lacM for *p*-coumaric acid and vanillin. The decolorization yields after 24 h of reaction of lacM with *p*-coumaric acid and vanillin were similar to those obtained by lacT. This indicates that the oxidized forms of *p*-coumaric acid and vanillin can efficiently decolorize melanin, although their oxidation rate by lacM was much lower than that by lacT. When lacM without a mediator was used for melanin decolorization during a sufficient reaction time, the decolorization yield was 5% after 24 h of reaction. The natural mediators, except vanillic acid, also act as more efficient mediators than HOBt for melanin decolorization by lacM after 24 h of reaction. When acetosyringone, syringaldehyde, and acetovanillone were used as mediators for lacM, the decolorization yields were 34%, 28%, and 31%, respectively, after 24 h of reaction. When vanillic acid was used as a mediator for both lacT and lacM, it showed the lowest decolorization yield. This may be caused by the low stability of the oxidized radical form of vanillic acid. Khammuang and Sarnthima reported that vanillin and vanillic acid could be used as mediators for melanin decolorization using laccase from *Lentinus polychrous* [28]. However, they showed much lower decolorization activity for melanin than acetosyringone when they were used as mediators for lacT and lacM. 

These results clearly indicate that natural mediators are more efficient for melanin decolorization by LMS than HOBt. HOBt has been considered as an efficient synthetic mediator for laccase because of its high redox potential and the catalytic role of the N-OH group of HOBt [5]. The efficiency of mediators to oxidize target substrates is highly dependent on the ability to form stable radicals as well as the steric hindrance caused by bulky alkyl substituents rather than the redox potential of the mediators [19,35]. The low stability of the oxidized intermediate of HOBt has been determined through cyclic voltammetry [6]. Therefore, the low decolorization yield by LMS using HOBt may be caused by the low stability of HOBt under the reaction conditions of laccase. Although the redox potential of syringaldehyde was lower than that of HOBt, syringaldehyde showed relatively higher stability than HOBt [6].

**Figure 2:**
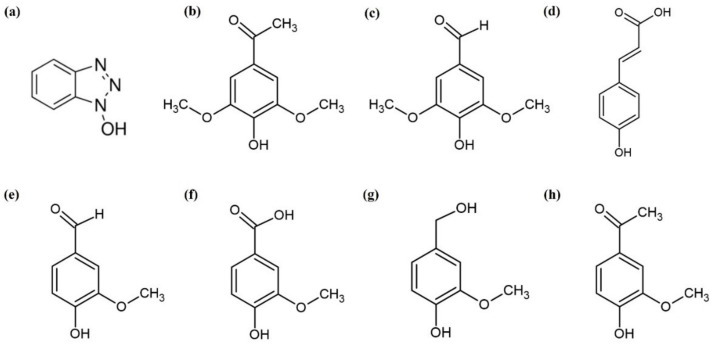
The structure of mediators used for LMS. (**a**) HOBt, (**b**) acetosyringone, (**c**) syringaldehyde, (**d**) *p*-coumaric acid, (**e**) vanillin, (**f**) vanillic acid, (**g**) vanillyl alcohol, (**h**) acetovanillone.

As shown in Figure 2, the natural mediators used in this work have various substituents (e.g., hydroxyl, methoxy, carboxyl, ketone, or aldehyde) at different positions on the benzene ring [12,19]. Mediators with two methoxy groups (acetosyringone and syringaldehyde) showed higher decolorization rates than those with one methoxy group. The decolorization rate obtained by *p*-coumaric acid with no methoxy group was dependent on the type of laccase. The *p*-coumaric acid with lacT showed a higher decolorization rate than those with one methoxy group, while *p*-coumaric acid with lacM showed the lowest decolorization rate in the 5 h reaction. Fillat et al. also showed similar results for the decolorization of flexographic inks by fungal laccases with natural mediators [36]. The phenolic natural mediators (acetosyringone, methyl syringate, and syringaldehyde) with two methoxy substituents in the ring were oxidized by laccase faster than *p*-coumaric acid with no methoxy group. This indicates that methoxy groups play a more important role as electron donors than the double bond of the lateral chain of *p*-coumaric acid. When the mediators with one methoxy group were compared, the decolorization yield increased in the following order: acetovanillone > vanillin > vanillyl alcohol > vanillic acid. Acetovanillone, which has a ketone group, showed a higher decolorization rate and yield than the mediators with aldehyde, hydroxyl, and carboxyl groups. Acetosyringone with a ketone group also showed a higher decolorization rate and yield than syringaldehyde with an aldehyde group.

In the following experiments, acetosyringone, syringaldehyde, and acetovanillone, which showed high melanin decolorization ability, were selected as mediators for LMS to decolorize melanin. The effect of the mediator on the decolorization reaction by LMS was investigated over time (Figure S1). The decolorization reaction using lacT with acetosyringone, syringaldehyde, and acetovanillone resulted in 21%, 18%, and 1% decolorization yields after 1 h of reaction, respectively. The decolorization reaction using lacM with acetosyringone and syringaldehyde resulted in 19% and 18% decolorization yields after 1 h of reaction, respectively. Both laccases showed similar reaction profiles when the same mediator was used. Acetosyringone and syringaldehyde significantly enhanced the decolorization rate during the initial reaction. These results show that acetosyringone and syringaldehyde containing dimethoxy groups were more efficient in enhancing the initial rate of decolorization by LMS than acetovanillone containing one methoxy group. Fillat et al. also reported that the methoxy groups in the ring structures of mediators act as accelerators for the oxidation of substrates [36]. On the other hand, the decolorization yield after 24 h of reaction by acetovanillone was similar to that by syringaldehyde, although acetovanillone moderately enhanced the reaction rate.

### 3.2. Effect of Mediator Concentration on Melanin Decolorization by LMS

**Figure 3:**
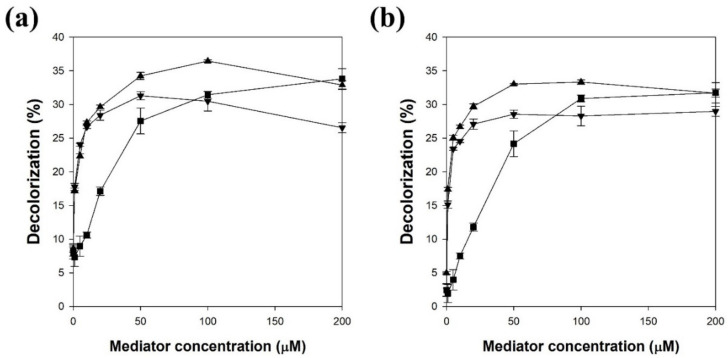
The effect of mediator concentration on the melanin decolorization by LMS using laccase from *T. versicolor* (**a**) and *M. thermophila* (**b**). ▲: acetosyringone, ▼: syringaldehyde, ■: acetovanillone. Reaction conditions: 50 μg/mL melanin and 0.1 M citric acid phosphate buffer (pH 5.5) at 25 °C for 24 h.

The effect of the mediator concentration on the decolorization yield by LMS was investigated (Figure 3). The LMS using lacT and 100 μM acetosyringone showed the highest decolorization yield, which was 4.4-fold higher than that without the mediator. The LMS using lacM and 100 μM acetosyringone showed the highest decolorization yield, which was 6.7-fold higher than that without the mediator. When lacT was used for LMS, the decolorization yields using acetosyringone, syringaldehyde, and acetovanillone increased with increasing concentration and then reached a maximum at concentrations of 100, 50, and 200 μM, respectively (Figure 3a). Acetosyringone and syringaldehyde more efficiently enhanced the decolorization yield at a lower concentration than acetovanillone. When lacM was used for LMS, the effect of mediator concentration was very similar to the result obtained with lacT. The decolorization yields using acetosyringone, syringaldehyde, and acetovanillone reached a maximum at concentrations of 100, 50, and 200 μM, respectively (Figure 3b). A mediator concentration of over 200 μM significantly decreased the decolorization yield (data not shown). Therefore, a mediator of 100 μM was chosen as the optimal concentration for the following experiments. Lloret et al. reported that the optimal mediator concentration should be used because laccase can be inactivated by a high concentration of mediator [6], whereas Khammuang and Sarnthima reported that the melanin decolorization activity of LMS using vanillin and vanillic acid was not significantly influenced by high concentrations of up to 10 mM [28]. The optimal mediator concentration may be dependent on the mediator type and the target compound of the laccase-catalyzed reaction [5,6]. When the LMS catalyzed the degradation of isoproturon, the degradation yield increased with increasing concentration of acetosyringone, while the concentrations of vanillin and syringaldehyde were not related to the degradation yield [7].

### 3.3. Cytotoxicity of Natural Mediators

**Figure 4:**
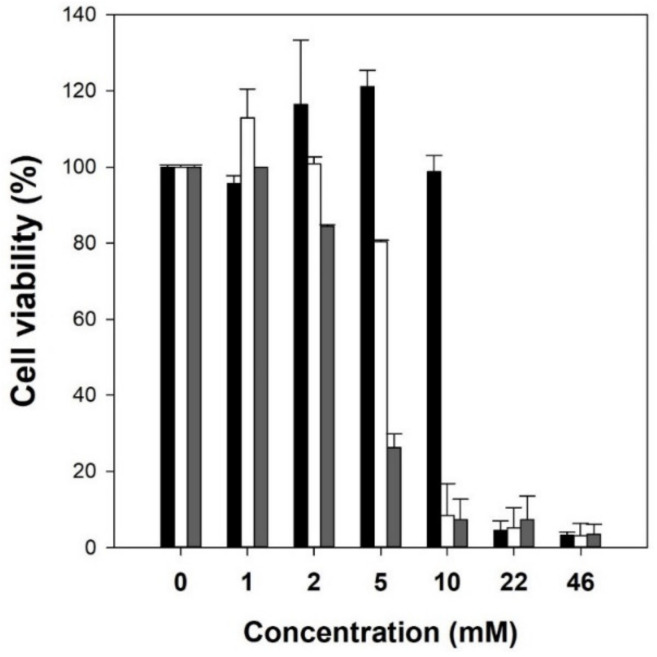
Cytotoxicity of natural mediators used for LMS. Black bars: acetosyringone, white bars: syringaldehyde, gray bars: acetovanillone.

To use natural mediators as skin-whitening cosmetic ingredients, the cytotoxicity of mediators (acetosyringone, syringaldehyde, and acetovanillone) was investigated using the B16F10 melanoma cell line. The natural mediators were treated on the cultured cells and the cell viability was measured by the NR assay. When mediators of over 22 mM were added to the cultured cells, they considerably reduced cell viability (Figure 4). When the concentration of the mediator was higher than 5 mM, cell viability increased in the following order: acetosyringone > syringaldehyde > acetovanillone. Furthermore, mediators of less than 1 mM showed no inhibitory effect on B16F10 melanoma cells. These results indicate that the optimal mediator concentration of 0.1 mM used in this work showed negligible cytotoxicity.

### 3.4. The Effect of pH on Melanin Decolorization by LMS

**Figure 5:**
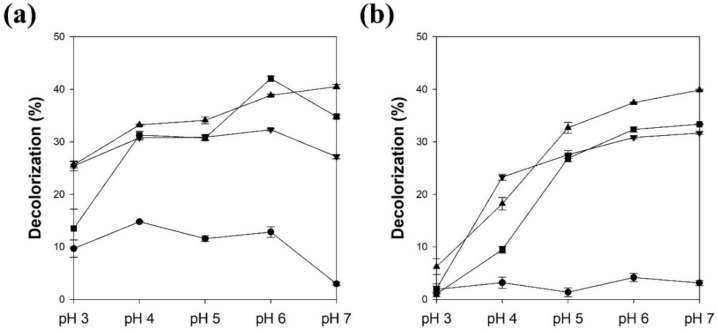
The effect of pH on melanin decolorization by LMS using laccase from *Trametes versicolor* (**a**) and *M. thermophila* (**b**). ●: no mediator, ▲: acetosyringone, ▼: syringaldehyde, ■: acetovanillone. Reaction conditions: 50 μg/mL melanin and 0.1 mM mediator at 25 °C for 24 h.

Figure 5 shows the effect of pH on the LMS decolorization yield. The lacT without a mediator showed the highest decolorization yield at pH 4, and the decolorization yield decreased with increasing pH (Figure 5a). This profile is similar to the effect of pH on laccase-catalyzed ABTS oxidation [37]. In general, lacT has optimal activity at acidic conditions of less than pH 5, and its activity decreases with an increase in pH. At higher pH, the hydroxide anions combine with the T2/T3 coppers of lacT and disturb the electron transfer and cause a decrease in catalytic activity. However, the effect of pH on LMS is more complicated and can be influenced by the activity and stability of laccase and oxidized mediators [1,7]. The decolorization yield of lacT with acetosyringone increased with increasing pH up to 7 (41%). The optimal decolorization yields of lacT with syringaldehyde and acetovanillone were 32% and 42%, respectively, at pH 6. Although lacT showed lower catalytic activity at higher pH, melanin decolorization increased with increasing pH. These results could be explained by the high activity and stability of the oxidized forms of natural mediators at high pH.

The lacM without a mediator showed the highest decolorization yield at pH 6, and lacM showed a lower decolorization yield in all pH ranges compared to lacT. The optimal pH of lacM was approximately 6 for the oxidation of ABTS [38]. Therefore, the optimal pH of lacM without a mediator was similar to that of lacM for ABTS oxidation. When the natural mediators were used with lacM, the decolorization yields were highly dependent on the reaction pH (Figure 5b). The decolorization yield of lacM with natural mediators increased with increasing pH up to pH 7. The maximum decolorization yields of lacM with acetosyringone, syringaldehyde, and acetovanillone were 40%, 32%, and 33%, respectively, at pH 7. The high melanin decolorization activity at neutral pH makes the use of LMS with natural mediators better for skin, because the optimal pH range is similar to that of normal skin (around 5.5).

### 3.5. Kinetic Study of Melanin Decolorization by LMS

A kinetic study of melanin decolorization by LMS with acetosyringone was investigated quantitatively to understand the enhancing effect of natural mediators (Table 1). The K_m_ value of lacT without a mediator was 10.6-fold higher than that of lacT with acetosyringone. This means that the affinity for melanin was highly enhanced by the use of a mediator. The k_cat_ value of lacT with the mediator was 22.6-fold higher than that of lacT without a mediator. This indicates that the decolorization rate was significantly increased by the mediator. The specificity constant (k_cat_/K_m_) of lacT was enhanced 247 times by using acetosyringone as a mediator. These results clearly show that the limited access of melanin to the active site of laccase was overcome by acetosyringone. The K_m_ value of lacM without a mediator was 2.4-fold higher than that of lacM with acetosyringone. The affinity for melanin can be enhanced by using a mediator. However, the increasing effect for melanin affinity by lacM with acetosyringone was lower than that by lacT with acetosyringone. The k_cat_ value of lacM was approximately 26% of that of lacT. The lacM without a mediator showed very low activity for melanin decolorization. However, the k_cat_ value of lacM with the mediator was 161-fold higher than that of lacM without a mediator. The decolorization rate by lacM was significantly increased by the mediator. Therefore, the specificity constant of lacM was 334 times enhanced using acetosyringone as a mediator. These results clearly demonstrate the usefulness of acetosyringone as a mediator of laccases for melanin decolorization.

### 3.6. Decolorization of the Melanin/Cellulose Film by LMS

**Figure 6:**
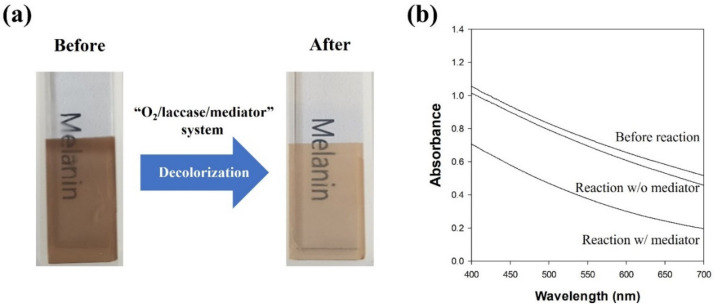
(**a**) Photo images and (**b**) spectrum of the melanin/cellulose films before and after the decolorization reaction by LMS using laccase from *M. thermophila* and acetosyringone.

A melanin/cellulose composite hydrogel film was prepared to mimic melanin in the skin. The melanin/cellulose hydrogel film could be prepared by the co-dissolution of melanin and cellulose in [Emim][Ac], which is followed by regeneration with water. The obtained film exhibited a transparent dark brown color. The decolorization of the melanin/cellulose hydrogel film was performed by lacM with acetosyringone in 0.1 M citric acid phosphate buffer (pH 5.5). The color of the melanin/cellulose film changed from dark brown to pale brown after the LMS-catalyzed reaction (Figure 6a). When the absorbance of the melanin/cellulose film was measured after the decolorization reaction in the range of 400–700 nm, the absorbance of the melanin/cellulose film decreased significantly over the entire wavelength range (Figure 6b). The decolorization of melanin was also confirmed through the measurement of color values (L*, a*, and b*) of the melanin/cellulose film using a colorimeter. The ΔL, ΔE, YI, and WI values were calculated from the color parameters (Table 2). After the melanin decolorization reaction by LMS, the L* (lightness) value of the melanin/cellulose film was considerably increased, while the a* (redness) and b* (yellowness) values decreased slightly. The ΔE value representing the color difference between the samples was 31.1. An ΔE value greater than 12 indicated that the colors of the film before and after the reaction are quite different from each other [31]. The yellowness (YI) of the film decreased from 209 to 92 after the decolorization reaction, while the whiteness (WI) increased from 16 to 43. Łopusiewicz et al. reported that the YI of poly(lactic acid)/melanin film increased with increasing melanin content, whereas the WI of the film decreased [32]. Therefore, the changes in the color properties quantitatively explain the decolorization of melanin in the melanin/cellulose hydrogel film. These results show that LMS can efficiently decolorize melanin in a cellulose hydrogel environment.

### 3.7. Decolorization of Natural Melanin Produced by Melanoma Cells

**Figure 7:**
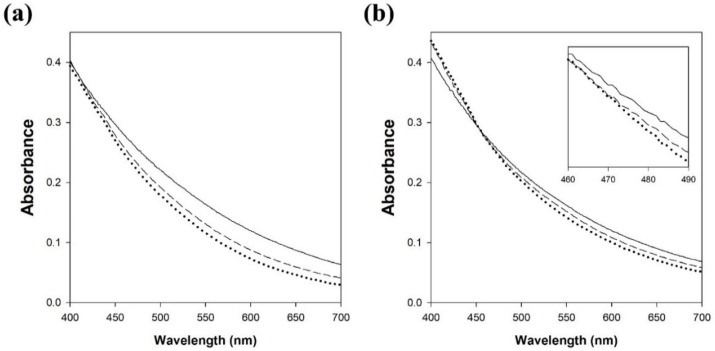
Changes in spectra of the synthetic melanin (**a**) and natural melanin (**b**) after the decolorization reaction by LMS using laccase from *M. thermophila* and acetosyringone. Solid line, dashed line, and dotted line represent no reaction, 15 min reaction, and 60 min reaction, respectively.

Natural melanin in human skin is divided into eumelanin (black to brown) and pheomelanin (yellow to red), while the synthetic melanin prepared by the oxidation of tyrosine with hydrogen peroxide is an analog of eumelanin. Therefore, the decolorization of natural melanin produced by melanoma cells was also investigated in this work. The decolorization of synthetic melanin and natural melanin by LMS with lacM and acetosyringone was compared by measuring the absorbance in the range of 400–700 nm (Figure 7). The absorbance of synthetic melanin rapidly decreased with increasing reaction time at all wavelengths. However, the absorbance of decolorized natural melanin showed a different pattern compared to that of decolorized synthetic melanin. The absorbance of decolorized natural melanin decreased at wavelengths greater than 450 nm, while the absorbance increased at wavelengths less than 450 nm. More studies are required to understand the increase in absorbance of decolorized natural melanin.

## 4. Conclusions

In this study, melanin decolorization was achieved by using the “O_2_/laccase/mediator” system, since laccase showed low catalytic activity for the direct oxidation of melanin due to its low redox potential and limited access of melanin into the active site of laccase. Seven kinds of natural mediators were successfully used to replace synthetic mediator (HOBt) for melanin decolorization by LMS using lacT and lacM. Among the tested natural mediators, acetosyringone and syringaldehyde, containing two methoxy groups, showed high decolorization rates and yields. Acetovanillone containing one methoxy group and one ketone group also showed a high decolorization yield in the equilibrium state. The LMS with natural mediators showed high decolorization activity at the pH of normal skin, and the cytotoxicity of natural mediators was very low. A kinetic study of LMS using acetosyringone for melanin decolorization showed that acetosyringone efficiently overcame the limitations of lacT and lacM by increasing the affinity for melanin and decolorization activity. LMS with acetosyringone decolorized the melanin present in the cellulose hydrogel film, which mimics skin. Furthermore, LMS with acetosyringone could decolorize not only synthetic eumelanin analogs prepared by the oxidation of tyrosine but also natural melanin produced by melanoma cells. Thus, LMS using natural mediators can be used as an effective skin-whitening agent in the cosmetics industry.

## Figures and Tables

**Table 1:** Kinetic constants of laccase from *T. versicolor* and *M. thermophila* for the melanin decolorization reaction using acetosyringone as a mediator.

		K_m_ (μg/mL)	k_cat_ (/h)	k_cat_/K_m_ (×10^−3^ mL/μg/h)
Laccase from *T. versicolor*	w/o mediator	284.6	0.44	1.5
	w/mediator	26.8	9.93	371.0
Laccase from *M. thermophila*	w/o mediator	319.7	0.11	0.4
	w/mediator	132.8	17.75	133.7

**Table 2:** Color parameters and indices of the melanin/cellulose hydrogel films before and after the decolorization reaction.

Decolorization of Melanin/Cellulose Film	L*	a*	b*	ΔL	ΔE	YI	WI
Before	28.3	13.3	41.4	-	-	208.9	16.2
After	59.2	12.2	38.0	30.9	31.1	91.6	43.0

## Data Availability

Not applicable.

## Supplementary Materials

The following are available online at https://www.mdpi.com/article/10.3390/polym13213671/s1, Figure S1: The melanin decolorization by LMS using laccase from *T. versicolor* and *M. thermophila*, Figure S2: The initial rate for the melanin decolorization reaction by laccase from *T. versicolor* and *M. thermophila*.
