# Supplementary material for: Laccase-Mediator System Using a Natural Mediator as a Whitening Agent for the Decolorization of Melanin
Source: Polymers (Basel). 2021 Oct 25;13(21):3671. doi: 10.3390/polym13213671 (PMC8587086; doi:10.3390/polym13213671)
Supplement: Supplementary file 1 [file polymers-13-03671-s001.zip › polymers-1431627-supplementary.pdf]

Supplementary

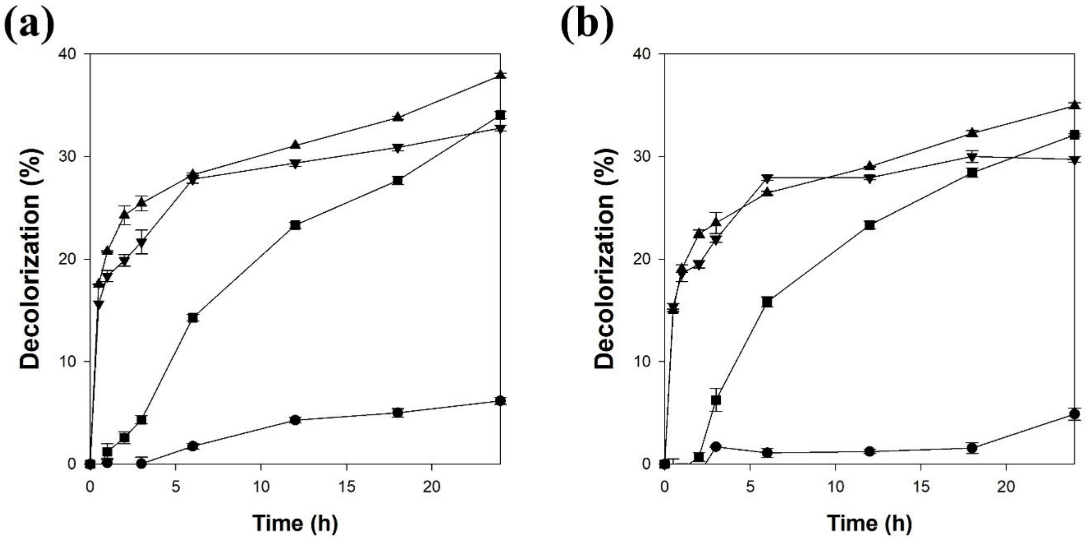

Figure S1. The melanin decolorization by LMS using laccase from *T. versicolor* (a) and *M. thermophila* (b). ●: no mediator, ▲: acetosyringone, ▼: syringaldehyde, ■: acetovanillone.

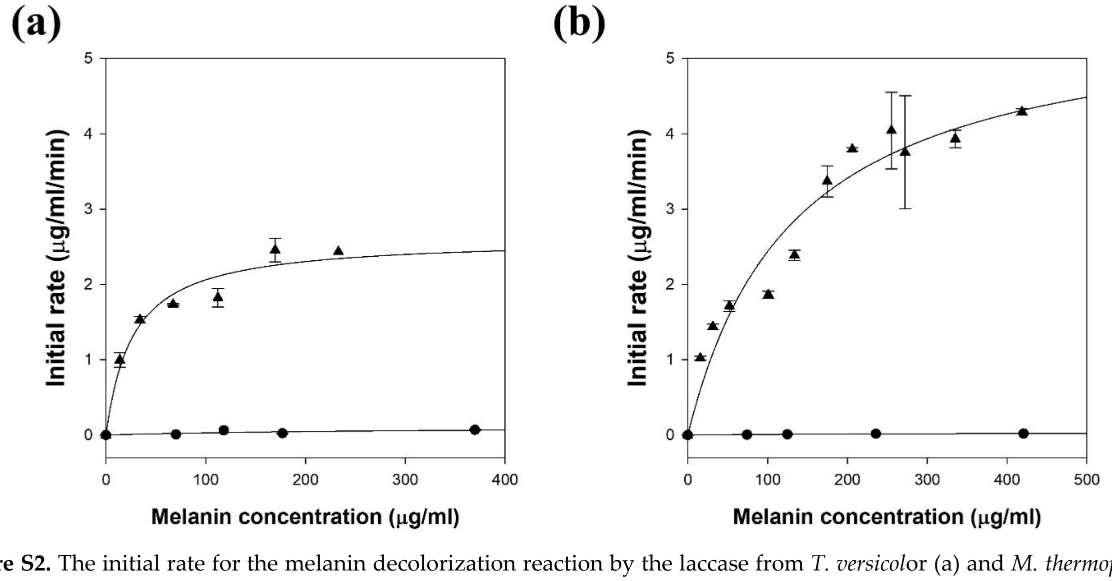

Figure S2. The initial rate for the melanin decolorization reaction by the laccase from *T. versicol*or (a) and *M. thermophila* (b). ●: no mediator, ▲: acetosyringone.
